# YRDC is a Prognostic‐Related Biomarker Correlated With Immune Infiltration and Drug Sensitivity in Pan‐Cancer

**DOI:** 10.1002/cnr2.70325

**Published:** 2025-09-02

**Authors:** Li Qiao, Yuetong Zhang, Pin Huang

**Affiliations:** ^1^ State Key Laboratory of Oncology in South China, Guangdong Key Laboratory of Nasopharyngeal Carcinoma Diagnosis and Therapy, Guangdong Provincial Clinical Research Center for Cancer Sun Yat‐sen University Cancer Center Guangzhou China; ^2^ Department of Rehabilitation Medicine Nanfang Hospital, Southern Medical University Guangzhou China; ^3^ Department of Intensive Care Unit (ICU) Sun Yat‐sen University Cancer Center Guangzhou China

**Keywords:** biomarker, drug sensitivity, immunotherapy, pan‐cancer, YRDC

## Abstract

**Background:**

YRDC has emerged as a potential biomarker in cancer, yet its prognostic value, oncogenic mechanisms, role in immune infiltration, and anticancer drugs efficacy in pan‐cancer remained poorly understood.

**Aims:**

This study aimed to comprehensively investigate YRDC's role in pan‐cancer and to explore the potential effects of YRDC on the immune infiltration pattern and anticancer drug sensitivity.

**Methods and Results:**

Based on bioinformatics analysis of multi‐omics data, we firstly demonstrated that YRDC was markedly overexpressed and associated with worse prognosis in various tumors. Further, our results indicated that genetic alterations, copy number variations, and methylation levels of YRDC might explain the different YRDC expression between tumors and controls. Additionally, YRDC might promote cancer via enhancing tumor cell proliferation, modulating immune cell infiltration, and enhancing drug resistance. Notably, YRDC emerged as a potential biomarker for predicting immunotherapy response and targeted drug efficacy.

**Conclusion:**

Our study identifies YRDC as a novel therapeutic target and a promising biomarker for cancer progression, immunotherapy response, and targeted drug sensitivity across pan‐cancers. These findings provide evidence for further research into YRDC's role in cancer biology and future clinical exercises.

**Abbreviations**
ACC: adrenocortical carcinoma
BLCA: bladder urothelial carcinoma
BRCA: breast invasive carcinoma
CESC: cervical squamous cell carcinoma and endocervical adenocarcinoma
CHOL: cholangiocarcinoma
CNVs: copy number variation
COAD: colon adenocarcinoma
ESCA: esophageal carcinoma
GBM: glioblastoma multiforme
HNSC: head and neck squamous cell carcinoma
ICIs: immune checkpoint inhibitors
KICH: kidney chromophobe
KIRC: kidney renal clear cell carcinoma
KIRP: kidney renal papillary cell carcinoma
LGG: brain lower grade glioma
LIHC: liver hepatocellular carcinoma
LUAD: lung adenocarcinoma
LUSC: lung squamous cell carcinoma
MESO: mesothelioma
OV: ovarian serous cystadenocarcinoma
PAAD: pancreatic adenocarcinoma
PCPG: pheochromocytoma and paraganglioma
PRAD: prostate adenocarcinoma
READ: rectum adenocarcinoma
SARC: sarcoma
SKCM: skin cutaneous melanoma
STAD: stomach adenocarcinoma
TFs: transcription factors
TGCT: testicular germ cell tumors
THCA: thyroid carcinoma
THYM: thymoma
UCEC: uterine corpus endometrial carcinoma
UCS: uterine carcinosarcoma
UVM: uveal melanoma
YRDC: yrdC N6‐threonylcarbamoltransferase domain containing protein

## 1 Introduction

Since the molecular hallmarks of cancers were extensively characterized, the therapeutic strategies of cancers have undergone a substantial transformation. The novel biomarkers, including immunomarkers such as PD‐L2 and PD‐L1, have precipitated a rapid evolution in cancer treatment [1]. Nonetheless, cancer remains the leading cause of mortality, responsible for more than 10.0 million deaths annually [2]. The identification of new key molecules could potentially unlock solutions to the pressing challenges posed by cancer.

The yrdC N6‐threonylcarbamoltransferase domain containing (*YRDC*) gene is a protein coding gene, which encodes YRDC protein. The YRDC protein is highly conserved with a dsRNA‐binding surface presenting from *E. coli* to *Homo sapiens* [3, 4]. The YRDC protein participates in N6‐threonylcarbamoyladenosine (t6A) formation in cytoplasmic and mitochondrial tRNAs [5]. The codon‐anticodon interaction and translational fidelity by the ribosomes were strengthened through modification of t6A [6, 7, 8]. YRDC is vital for protein translation at the level of codon recognition.

YRDC was believed to play a critical role in the occurring development of cancers [9]. YRDC can promote the proliferation of cancer cells, and it was considered to be a proliferation‐associated protein [9]. The promotion of hepatocellular carcinoma cell proliferation was thought to be a result of YRDC's positive effect on MEK/ERK signaling pathways [10]. According to Shen et al.'s study, inhibition of YRDC could potentially hinder the progression of non‐small cell lung cancer [9]. However, the expression of YRDC in more types of tumors and its mechanisms involved in tumorigenesis and progression remain unclear.

Previous studies have shown that YRDC could affect lenvatinib sensitivity via the RAS/RAF/MEK/ERK pathway in hepatocarcinoma [11]. As we know, the RAS/RAF/MEK/ERK pathway is vital for tumor proliferation, survival, and differentiation [12]. More and more inhibitors of the RAS/RAF/MEK/ERK pathway were found [13]. The potential impact of YRDC on the efficacy of other antitumor drugs remains to be elucidated.

Immune checkpoints are a type of cell surface receptor that regulate immune pathways, and their activation has been linked to tumor growth inhibition [14]. Immune checkpoint inhibitors (ICIs) work by blocking immunosuppressive pathways, then reversing the immunosuppressive tumor microenvironment. Currently, immunotherapy has developed rapidly and has become one of the most effective therapies for cancer. In lung cancer, on both a second‐ and first‐line basis, ICIs are more effective than chemotherapy alone [15]. Despite the significant benefits offered by immune checkpoint inhibitors, only a subset of patients can respond to these treatments [16]. Predictors of response in cancer patients treated with ICIs have driven great interest. Several studies demonstrated the RNA expression of key molecular biomarkers can serve as biomarkers for ICI treatment [16]. Sun et al. showed that immune checkpoint inhibitors can only work in lung carcinomas with specific TP53 subtypes [17]. Other studies showed that the expression level of PD‐L1 could predict the response to immune checkpoint inhibitors [18]. However, these indicators have displayed a limited ability to anticipate the therapeutic efficacy of ICIs. More efforts are needed in order to find novel, effective markers to predict the outcomes of cancer patients with ICI treatment. Thus, we explored the relationships between YRDC expression and immunity infiltration. Whether YRDC predicts immunotherapy efficacy was also assessed in the study.

In recent years, studies of pan‐cancer have emerged as a powerful tool to identify both commonalities and distinctions in tumor biology [19, 20]. By integrating data across diverse malignancies, the comprehensive approach not only aided in uncovering shared pathways but also facilitated the development of more generalized treatment strategies. For instance, recent studies have demonstrated significant potential in identifying novel biomarkers and therapeutic approaches through pan‐cancer analysis [21, 22].

In conclusion, previous studies have shown that YRDC promoted the proliferation of certain carcinoma cells and had effects on lenvatinib sensitivity. However, whether YRDC expression was a common mechanism of tumorigenesis and progression at the pan‐cancer level was still unclear. It would be helpful to figure out the oncogenic mechanisms of YRDC and whether YRDC had effects on other drug resistance or immunotherapy response. Thus, we performed a comprehensive bioinformatics analysis with multi‐omics data to show the expressions and prognostic values of YRDC in pan‐cancer. Then we explored the *YRDC* oncogenic mechanisms through analyzing gene mutation, methylation analysis, related transcription factors, and coexpression gene networks of *YRDC*. Lastly, the relationship between YRDC and immunotherapy efficacy and anticancer drug sensitivity was assessed. This study will help us more clearly understand the role of YRDC in cancer and lay some groundwork for future research to validate and elucidate the mechanisms of YRDC in tumorigenesis and anticancer drug efficacy.

## 2 Materials and Methods

### 2.1 Data Collection and Expression Analysis

**FIGURE 1:**
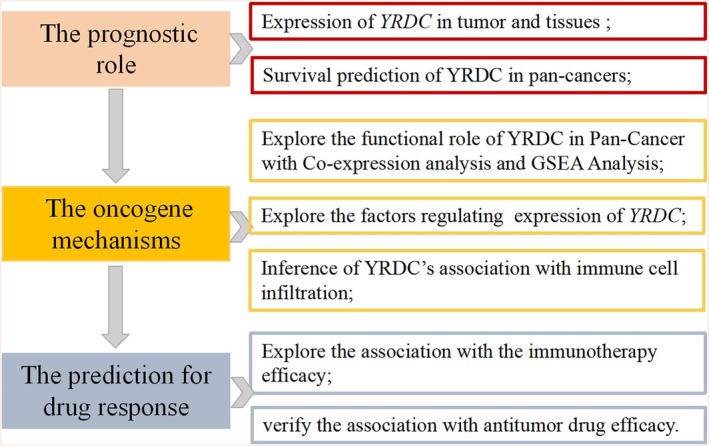
The schematic diagram illustrates the workflow of this study.

The overarching analytical workflow of this study is depicted in Figure 1. We obtained the RNA‐seq data (TCGA cohort) and associated clinical data from UCSC Xena (http://xena.ucsc.edu/) [23]. We examined RNA expression of YRDC in numerous cell types based on single‐cell RNA‐seq data using the “Gene” module of TISCH (http://tisch.comp‐genomics.org/search‐gene/) [24].

In our study, 31 solid tumors were examined, including: adrenocortical carcinoma (ACC), bladder urothelial carcinoma (BLCA), breast invasive carcinoma (BRCA), cervical squamous cell carcinoma and endocervical adenocarcinoma (CESC), cholangiocarcinoma (CHOL), colon adenocarcinoma (COAD), esophageal carcinoma (ESCA), glioblastoma multiforme (GBM), head and neck squamous cell carcinoma (HNSC), kidney chromophobe (KICH), kidney renal clear cell carcinoma (KIRC), kidney renal papillary cell carcinoma (KIRP), brain lower grade glioma (LGG), liver hepatocellular carcinoma (LIHC), lung adenocarcinoma (LUAD), lung squamous cell carcinoma (LUSC), mesothelioma (MESO), ovarian serous cystadenocarcinoma (OV), pancreatic adenocarcinoma (PAAD), pheochromocytoma and paraganglioma (PCPG), prostate adenocarcinoma (PRAD), rectum adenocarcinoma (READ), sarcoma (SARC), skin cutaneous melanoma (SKCM), stomach adenocarcinoma (STAD), testicular germ cell tumors (TGCT), thyroid carcinoma (THCA), thymoma (THYM), uterine corpus endometrial carcinoma (UCEC), uterine carcinosarcoma (UCS), uveal melanoma (UVM).

### 2.2 Statistical Analysis of Survival

The survival analysis was restricted to solid tumors with complete clinical information. We analyzed the prognostic relevance of YRDC using univariate Cox regression across cancer types. Multivariate Cox regression analysis was conducted to identify independent prognostic factors. In this process, the surv_cutpoint function from the R package “survminer” was utilized to achieve the optimal cutoff value of YRDC expression level.

### 2.3 Gene Mutation and Methylation Analysis of YRDC and Related Transcription Factors Identification

The genetic alteration status of YRDC was assessed using cBioPortal (https://www.cbioportal.org/, based on TCGA Pan‐Cancer Atlas data) [25, 26]. Correlations between CNV and YRDC RNA expression levels were evaluated by MEXPRESS (https://mexpress.be/) [27, 28]. We analyzed the methylation profiles of tumor tissues and normal tissues using Oncodb (http://oncodb.org/) [29]. To determine which transcription factors (TFs) are most likely to enhance the expression of YRDC, we utilized the DbToolkit (http://dbtoolkit.cistrome.org/) [30, 31].

### 2.4 Co‐Expression and Functional Enrichment

To construct the co‐expression networks of YRDC in pan‐cancer, we investigated the co‐expressed genes for YRDC by calculating Spearman's rank correlation coefficient with all other genes in each cancer. Benjamini‐Hochberg was used as a *p* value correction method to adjust for the multi‐comparison. The fgsea package (https://github.com/ctlab/fgsea) was utilized to perform GSEA analysis [32] focusing on Hallmark's gene sets, downloaded from the Sigdb database (https://www.gsea‐msigdb.org/gsea/msigdb/) [33, 34]. We defined the gene sets with P values lower than 0.05 by GSEA as correlated pathways.

### 2.5 Evaluation of the Function of YRDC Using CRISPR Screens

We obtained CRISPR screen data from the BioGRID (https://orcs.thebiogrid.org/) [35]. For each dataset, phenotype‐related genes were defined according to the authors' choice. In short, those studies with a HIT value of yes are considered to support the conclusion that the YRDC was correlated to the phenotype (cell proliferation or drug resistance).

### 2.6 Characterizing the Tumor‐Infiltrating Immune Cells

The proportion of tumor infiltrating immune cells (TILs) in each TCGA tumor sample was estimated by using the CIBERSORT function (https://cibersort.stanford.edu/) [36, 37]. The relationship between YRDC expression and the proportion of TILs was assessed by calculating Spearman correlation coefficients for them.

### 2.7 Assessment of YRDC as a Predictor of Immunotherapy

The immune checkpoint blockade (ICB) therapy cohort provided by Liu et al. was used to assess the predictive value of YRDC to immunotherapy [38]. We obtained transcriptomic and clinical data for this cohort from dbGaP (phs000452).

### 2.8 Drug Sensitivity Analysis

We obtained the half maximal inhibitory concentration (IC50) data for multiple cancer cell lines as well as their associated RNA‐seq data from the CellMiner database (https://discover.nci.nih.gov/cellminer/) [39, 40]. We assessed the relationship between YRDC expression and drug sensitivity by calculating Spearman's rank correlation coefficients for them.

### 2.9 Statistics Analysis

All data analysis was carried out using R (version 4.1.0) unless otherwise stated. The tumors that were subjected to each analysis are described in Table S1.

## 3 Results

### 3.1 Significantly Higher Expression of YRDC in a Variety of Tumor Cells by Pan‐Cancer Analysis

**FIGURE 2:**
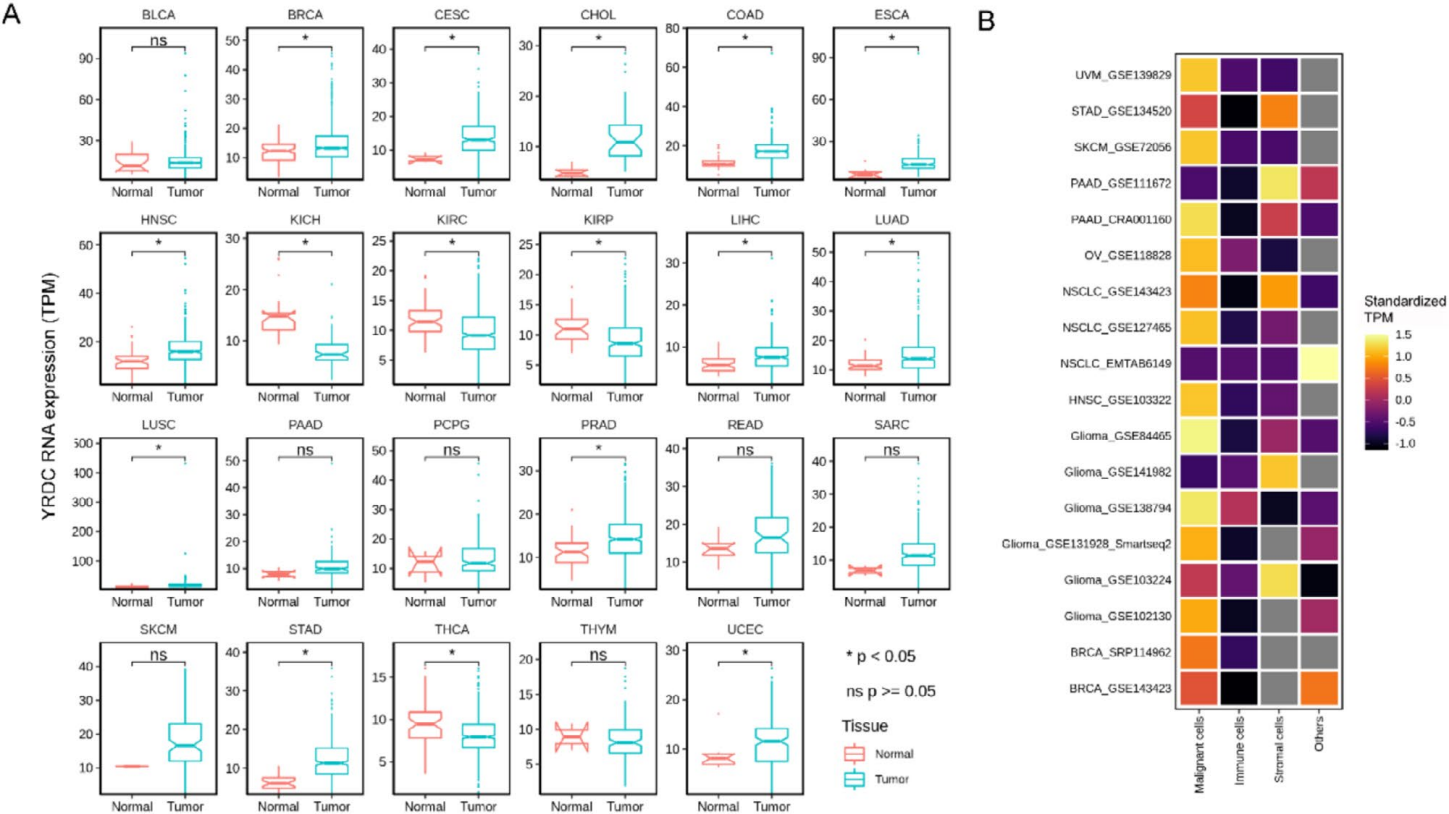
RNA expression of YRDC in different types of cancer. (A) The RNA expression of YRDC in different types of cancers. (B) The RNA expression in different cell types of multiple cancers. TPM values were standardized in each tumor. NSCLC, non‐small‐cell lung carcinoma.

Firstly, we compared the differences in mRNA expression of *YRDC* in solid tumors and their matched normal tissues. YRDC was highly expressed in 12 of 23 available solid tumor tissues (*p* < 0.05, Wilcoxon test, Figure 2A), including breast invasive carcinoma (BRCA), cholangiocarcinoma (CHOL), colon cancer (COAD), esophageal carcinoma (ESCA), head and neck squamous cell carcinoma (HNSC), liver hepatocellular carcinoma (LIHC), lung adenocarcinoma (LUAD), lung squamous cell carcinoma (LUSC), prostate adenocarcinoma (PRAD), stomach adenocarcinoma (STAD), and uterine corpus endometrial carcinoma (UCEC). There were four of 23 tumor tissues with decreased YRDC RNA levels (*p* < 0.05, Wilcoxon test, Figure 2A), including Kidney Chromophobe (KICH), Kidney renal clear cell carcinoma (KIRC), Kidney renal papillary cell carcinoma (KIRP), and Thyroid carcinoma (THCA).

Secondly, using the TISCH database, the cellular expression level of YRDC was relatively higher in tumor cells than in other cell types (e.g., immune cells, stromal cells, etc.) in uveal melanoma (UVM), STAD, skin cutaneous melanoma (SKCM), ovarian serous cystadenocarcinoma (OV), HNSC, and BRCA. As for pancreatic adenocarcinoma (PAAD), NSCLC, and glioma, several cohorts verified that tumor cells expressed much more YRDC (as seen in Figure 2B). In other words, *YRDC* was highly expressed in various tumor tissues. Moreover, almost all available data have shown that YRDC was mainly expressed in tumor cells. It indicated that YRDC might contribute to oncogenesis.

### 3.2 YRDC Serving as a Prognostic Marker for Multiple Cancers

**FIGURE 3:**
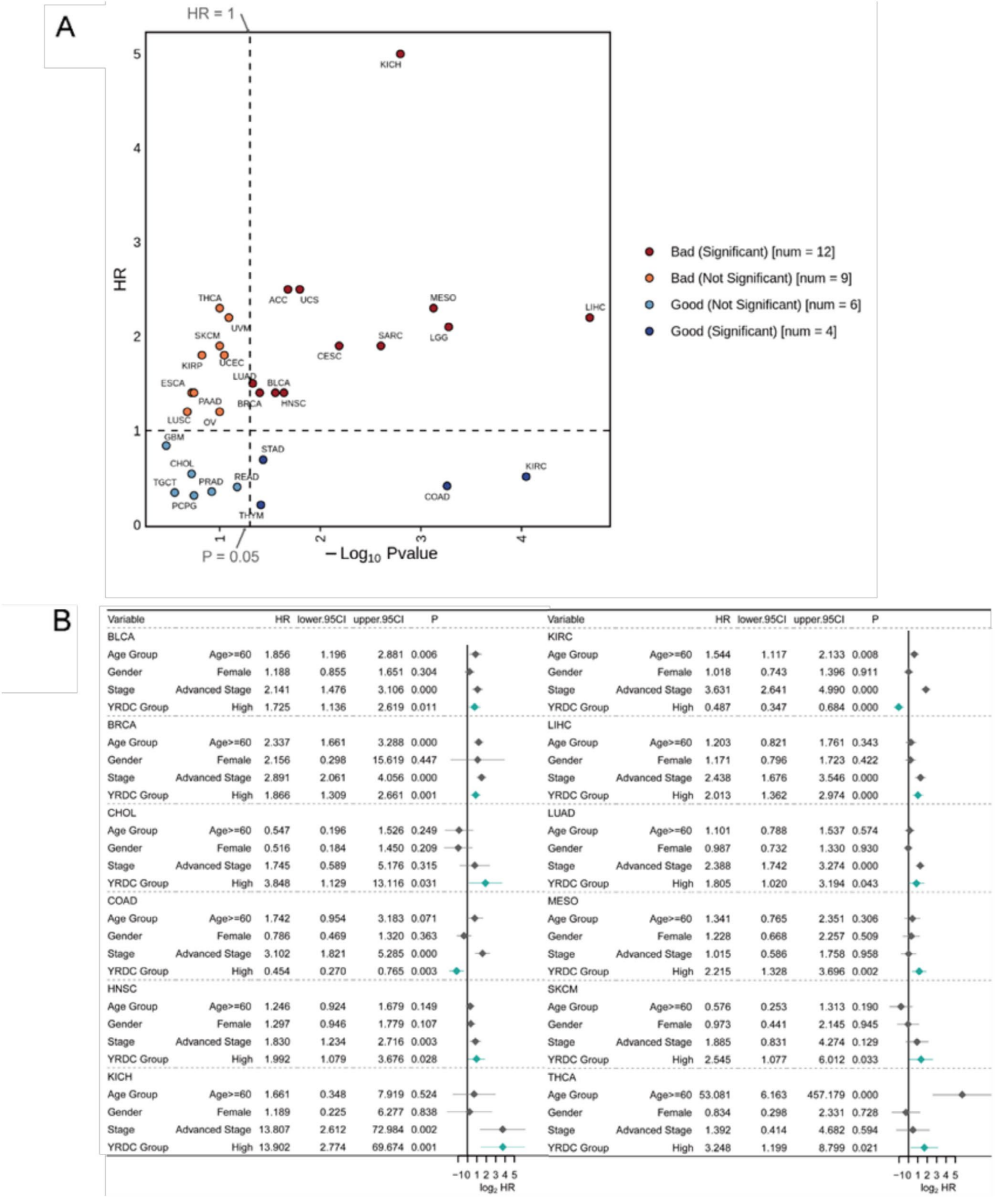
The prognostic value of YRDC in a variety of tumors. (A) The survival analysis results of patients with different *YRDC* expression. (B) The results of multivariate Cox regression for multiple tumors.

Nearly no significant differences in *YRDC* expression levels between tumor patients of different age groups, gender, or stage were observed (Figure S1). We assessed the effect of YRDC on the prognosis of patients in 31 solid tumors. We observed a significantly worse prognosis in patients with high *YRDC* expression compared to those with low *YRDC* expression in 12 tumor patients including adrenocortical carcinoma (ACC), LUAD, BRCA, bladder urothelial carcinoma (BLCA), HNSC, uterine carcinosarcoma (UCS), cervical squamous cell carcinoma and endocervical adenocarcinoma (CESC), sarcoma (SARC), mesothelioma (MESO), brain lower grade glioma (LGG), kidney chromophobe (KICH), LIHC (*p* < 0.05, univariate Cox regression, Figures 3A and S2). Otherwise, a better prognosis was observed in patients with high YRDC expression compared to patients with low YRDC expression in only four tumors, including thymoma (THYM), STAD, COAD, and kidney renal clear cell carcinoma (KIRC) (*p* < 0.05, univariate Cox regression, Figures 3A and S2). We further adjusted for other clinical risk factors like age, gender, and clinical stage; YRDC was considered an independent prognostic marker in 12 tumors (*p* < 0.05, multivariate Cox regression, Figure 3B), and in 10 among 12 tumors (BLCA, BRCA, CHOL, HNSC, KICH, LIHC, LUAD, MESO, SKCM and THCA), high *YRDC* expression predicted a worse prognosis for patients (Figure 3B). In COAD and KIRC patients, high expression of YRDC could independently predict better prognosis. It suggested that YRDC might be associated with disease progression in multiple tumors and be used as a prognostic marker for patients with multiple tumors.

### 3.3 Oncogenic Mechanisms of YRDC

#### 3.3.1 Genomic Changes Regulating Expression of YRDC

**FIGURE 4:**
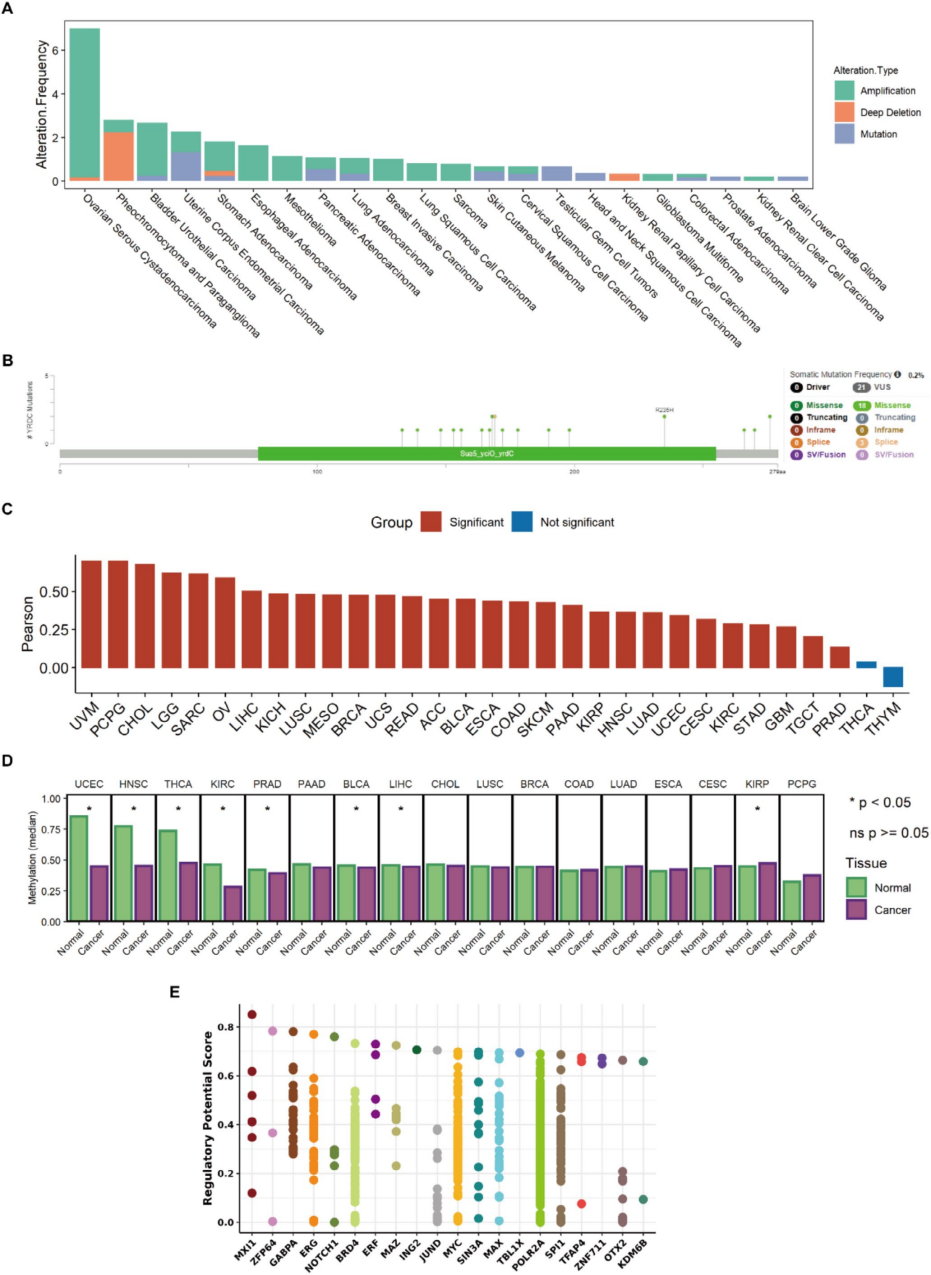
Gene mutations, methylation levels, and related transcription factors of YRDC. (A) Gene alteration frequency analysis of *YRDC*. (B) Sites of different mutation types of *YRDC*. (C) Correlations of CNV and YRDC RNA expressions in different tumors. (D) The DNA methylation levels of *YRDC* in tumors and normal tissues. The statistics were provided from the Oncodb database. (E) Potential transcription factors of *YRDC*. Dots on a single‐axis line indicate the same factor.

Genomic alterations can cause changes in gene expression and vary the function of genes. We characterized the genomic and epigenetic features of YRDC in tumors. First, we analyzed genetic alterations of *YRDC*, including mutations, amplifications, and deep deletions. We observed that the major genotypic alterations in YRDC were amplification and mutation in a variety of cancers (Figure 4A). Further, we found that the major *YRDC* mutation types were missense mutations and a large number of mutations were concentrated in functional structural regions (Figure 4B). These mutations may make *YRDC* have altered RNA expression or protein structure. The copy number variation (CNVs) alteration may change gene expression levels. Afterward, we checked whether *YRDC* RNA expression correlates with CNV. Our results showed that *YRDC* RNA expression was significantly correlated with CNV in a variety of tumors (Pearson correlation coefficient > 0.2 and *p* < 0.05, Figure 4C), suggesting that the increased CNV may be responsible for the high *YRDC* expression in tumor tissues. Methylation levels of genes can cause changes in RNA expression levels. Hypermethylation of DNA decreases RNA expression, and conversely, hypomethylation of DNA may increase RNA expression. Therefore, we compared the methylation levels of YRDC genes in tumor tissues and normal tissues by using Oncodb (as shown in Methods). We observed that in seven tumors, tumor tissues had lower DNA methylation levels compared to normal tissues (*p* < 0.05, Figure 4D), which we did not observe in other tumors. This suggested that the aberrant hypomethylation of DNA may also be responsible for the high *YRDC* expression in these tumors. Finally, we screened several transcription factors that may regulate YRDC using tools. The top 20 factors were illustrated in Figure 4E.

#### 3.3.2 YRDC Enhancing Tumor Cell Proliferation With Co‐Expression Analysis and CRISPR Screening Data

**FIGURE 5:**
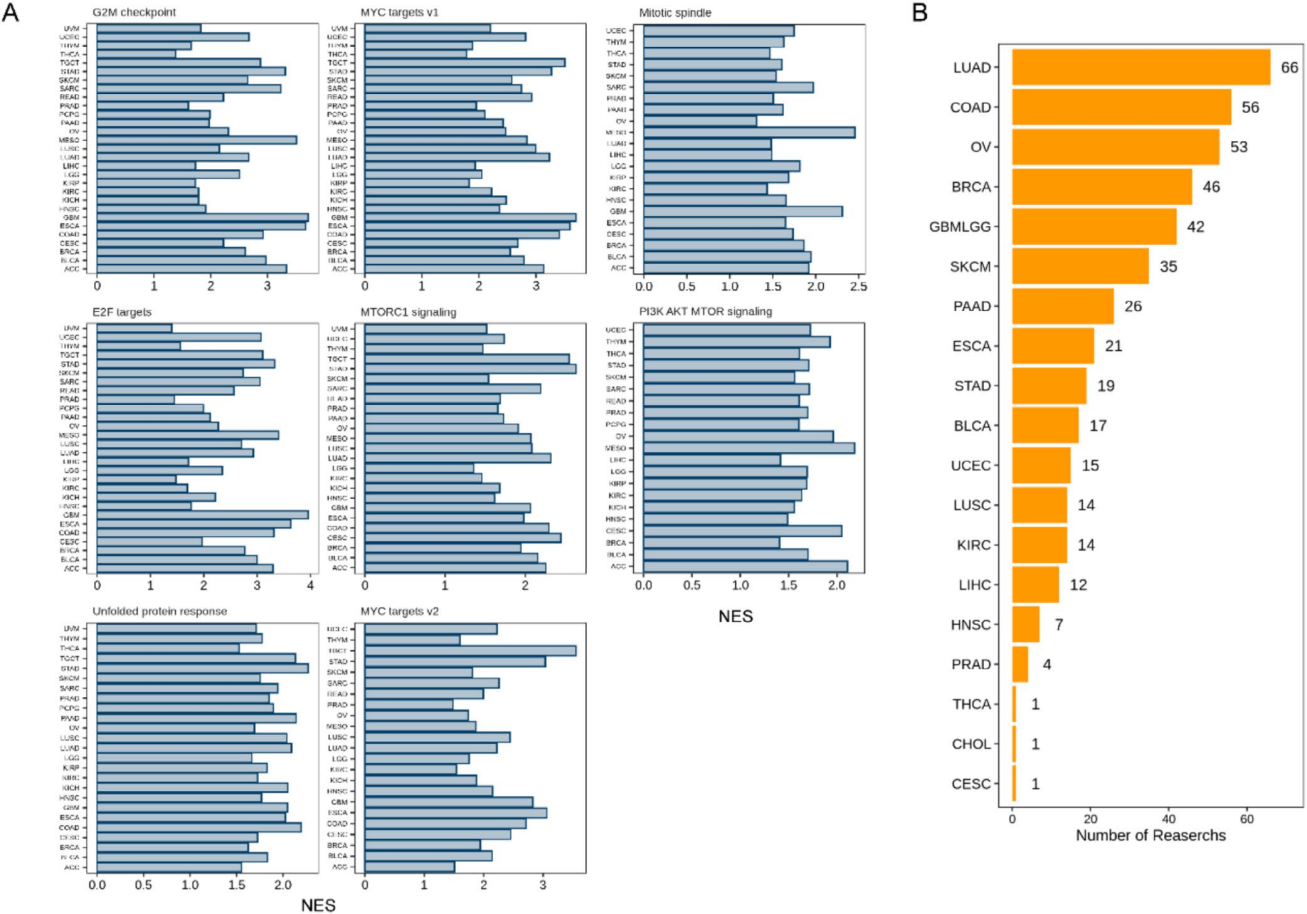
High level of YRDC boosting cell proliferative ability. (A) The GSEA enrichment results of YRDC in different types of cancers. Only pathways significantly enriched based on the GSEA analysis (*p* < 0.05) were shown. (B) CRISPR Screens studies verified YRDC could accelerate the proliferation of cancer cells. The X‐axis indicates the number of CRISPR Screen studies demonstrating the involvement of this gene in the proliferation of tumor cell lines. The Y‐axis displays the types of tumors studied in CRISPR Screens.

We further elucidated its potential cancer‐promoting mechanisms through functional annotation. We constructed YRDC co‐expression gene networks in each tumor data, and based on which, a GSEA analysis was further performed to explore the function of YRDC (as shown in Methods). YRDC was associated with multiple cell proliferation‐related pathways among numerous cancers (including G2M checkpoint, MYC Targets (V1 and V2), and E2F targets, see Figure 5A) [34]. This suggested that YRDC might have a mitogenic effect. Then, publicly available CRISPR Screening data was used to determine whether *YRDC* can promote tumor cell proliferation. CRISPR screens, which can provide high‐quality evidence, can be used to study phenotype‐related genes [41]. According to our results, 450 CRISPR Screens studies in 19 tumors (including LUAD, COAD, OV, BRCA, GBM, LGG, SKCM, PAAD, ESCA, STAD, BLCA, UCSE, LUSC, KIRC, LIHC, HNSC, PRAD, THCA, CHOL and CESC) identified YRDC as an essential gene for tumor cell proliferation (Figure 5B).

### 3.4 YRDC Related to Tumor Immune Environment and Immunotherapy

**FIGURE 6:**
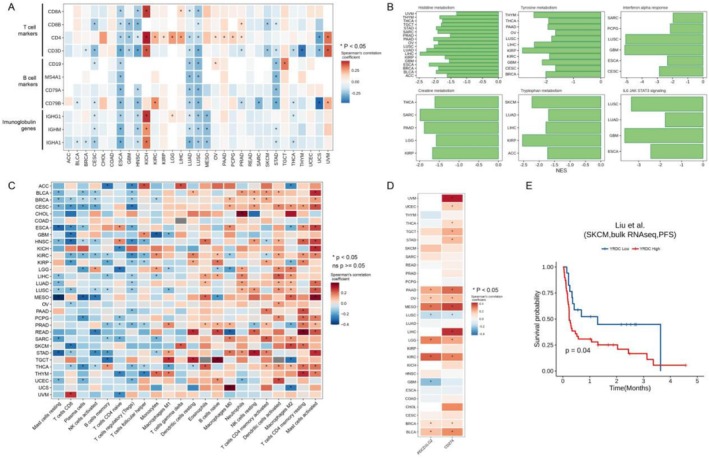
Relationship of YRDC and tumor immune features. (A) Correlation of *YRDC* RNA expression with cellular markers and immunoglobulin genes. (B) GSEA enrichment results of YRDC in multiple tumors. Only pathways that were deemed significantly enriched based on the GSEA analysis (*p* < 0.05) were shown in the figure. (C) Correlation of *YRDC* RNA expression with the proportion of tumor‐infiltrating immune cells. (D) Correlation of RNA expression of YRDC with PD‐L1/PD‐L2. (E) Kaplan–Meier plots verify the prognostic ability of YRDC in patients treated by immunotherapy.

In the gene co‐expression network, we observed that YRDC was associated with the expression of multiple immune cell signature genes at the pan‐cancer level, including T‐cell signature genes (*CD3D*, *CD4*, *CD8A, CD8B*), B‐cell signature genes (*CD19, MS4A1*), and multiple immunoglobulin genes (Figure 6A). Meanwhile, in GSEA results, YRDC was related to the immunologic pathway (including interleukin 6 (IL6)‐The Janus kinase (JAK)‐the signal transducer and activator of transcription 3 (STAT3) signaling, interferon‐alpha (IFN‐α) response) (Figure 6B). It is suggested that the expression level of YRDC may be involved in immune cell infiltration in the tumor microenvironment. Therefore, CIBERSORT was used to resolve the proportion of immune cells in TCGA pan‐cancerous tissue samples. We observed that *YRDC* expression levels were inversely correlated with CD8+ T cells, memory B cells, plasma cells, and activated NK cells in most of the tumor tissues (Figure 6C). Thu, YRDC might have effects on the infiltration of multiple immune cells. We also examined the correlation of the expression of *YRDC* with the expression of immune checkpoints PD‐L1 (*CD274*) and PD‐L2 (*PDCD1LG2*). Both *PD‐L1* and *PD‐L2* were correlated with *YRDC* expression at the pan‐cancer level (Figure 6D).

Further, we explored the role of *YRDC* in immunotherapy. Patients with higher *YRDC* expression had significantly shorter PFS after anti‐PD‐1 monoclonal antibody treatment than those with low expression (Figure 6E). In other words, *YRDC* might be associated with poor anti‐PD‐1 monoclonal antibody treatment efficacy and be served as a prognostic biomarker for immunotherapy.

### 3.5 YRDC Associated With Antitumor Drug Efficacy

**FIGURE 7:**
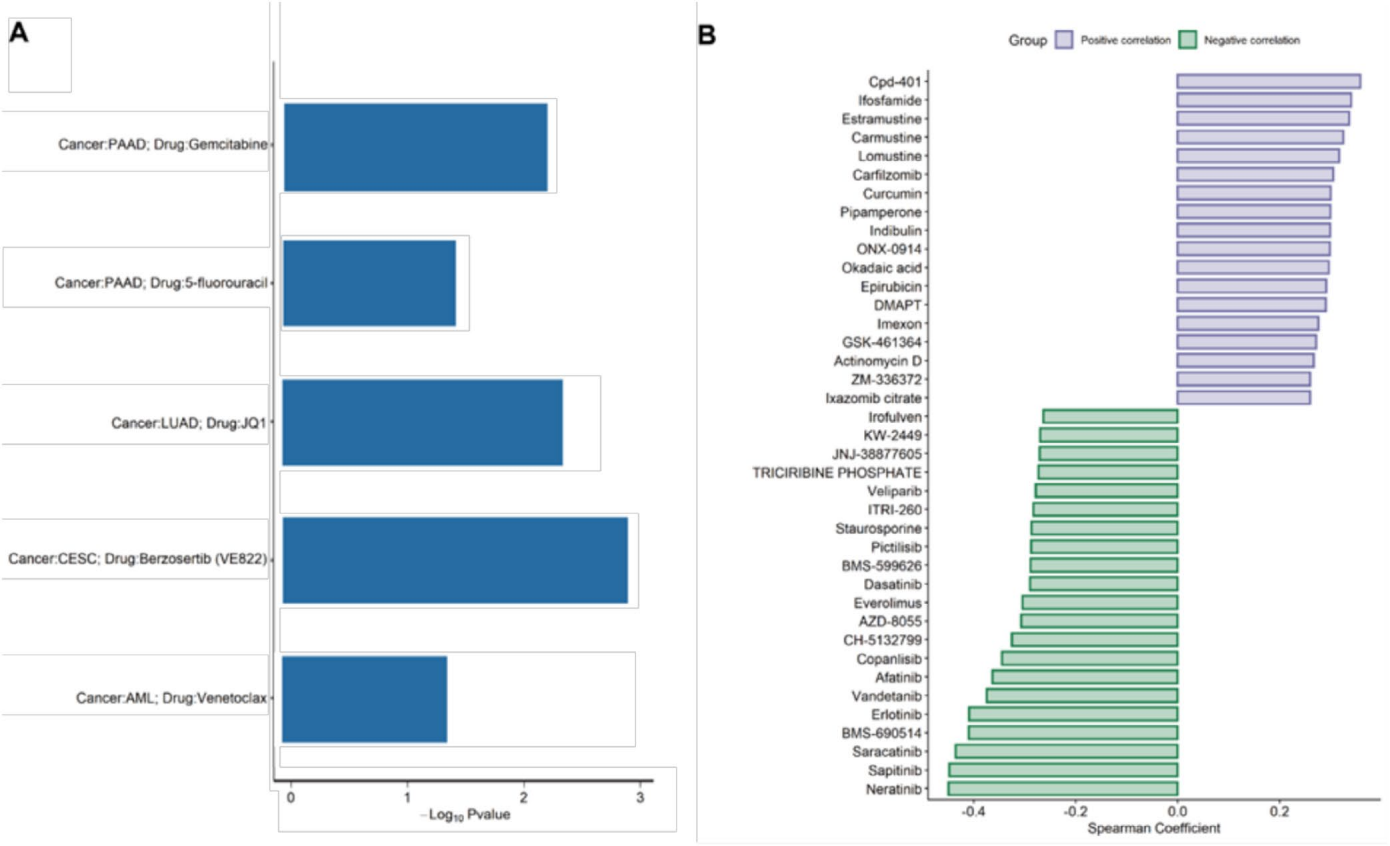
YRDC exacerbating drug resistance. (A) CRISPR Screens studies confirmed that YRDC was associated with drug resistance. The Y‐axis indicates the tumor type and the drugs. The X‐axis shows the logarithm of the negative *p* value of YRDC in each CRISPR Screens study. (B) The bar plot shows the Spearman correlation coefficient of *YRDC* RNA expression and drug IC50.

Our previous results showed that YRDC was associated with the unfolded protein response (Figure 7A), suggesting that YRDC may be associated with tumor drug resistance. Next, five relevant studies suggested that *YRDC* is associated with drug resistance using public CRISPR Screens (Figure 7A), including resistance to Gemcitabine, fluorouracil, JQ1, Benzosertib, and venetoclax. To further explore the possibility of *YRDC* resistance, the correlations of IC50 data of 400 FDA‐approved or clinically available tumor‐related drugs with *YRDC* expression were conducted. Higher IC50 indicates more tolerant cells to the drug. Five drugs showed significant correlations between IC50 and *YRDC* expression in the study (*p* < 0.05). Positive correlations of *YRDC* with the IC50 of Cpd‐401, Ifosfamide, and Estramustine et al. (Spearman correlation coefficients > 0.2 and *p* < 0.05 for all, Figure 7B). It is suggested that *YRDC* may be associated with resistance to these drugs. Meanwhile, we observed that the IC50 of Neratinib, Sapitinib, and Saracatinib was negatively correlated with the expression of *YRDC* (Spearman correlation coefficients < −0.2 and *p* < 0.05, Figure 7B). It is suggested that tumors with high *YRDC* expression might be sensitive to these drugs.

## 4 Discussion

**FIGURE 8:**
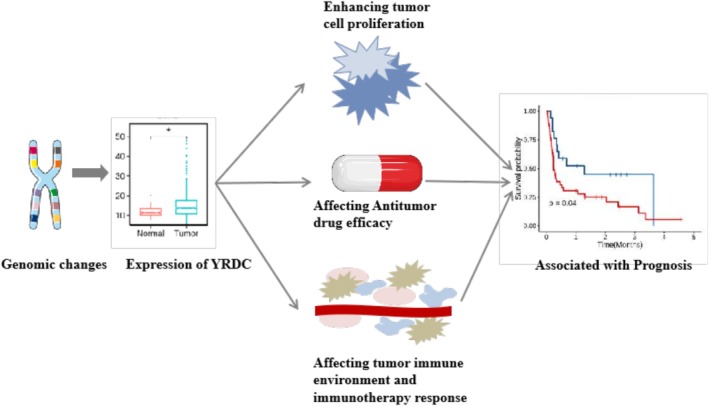
Flowchart depicting the study's main finding.

The comprehensive pan‐cancer analysis provides a broad perspective on YRDC's role across multiple cancer types. As Figure 8 shows, the mutation, methylation, or related transcription factors of YRDC could change YRDC expression; Then high YRDC expression upregulated tumor cell proliferation and changed the immune cell infiltration in tumors by affecting the expression of cytokines or immune proteins such as IFN‐α, PD‐L1/PD‐L2, etc. Furthermore, the positive relationships between YRDC and immunotherapy efficacy and several anticancer drugs sensitivity were confirmed. These firstly provide evidence for YRDC as a prognostic and drug sensitivity marker in pan‐cancer. This exploration is pivotal to finding new therapeutic and prognostic strategies that could improve current treatments.

In this study, we found that YRDC was highly expressed in 17 of 28 available solid tumors. Moreover, osteosarcoma highly expressed YRDC as Gong et al. shown [42]. YRDC was firstly verified to be overexpressed in tissues of BRCA, CHOL, COAD, ESCA, HNSC, PRAD, STAD, and UCEC based on the TCGA cohort. According to data from the TISCH database, it was firstly reported that YRDC expression was relatively higher in tumor cells than non‐tumor cell types in UVM, STAD, SKCM, OV, HNSC, BRCA, PAAD, NSCLC, and glioma. In BLCA, READ, and SARC, higher expression of YRDC was observed in tumor tissues than in normal tissues. Using the public CRISPR screening data, which is reliable experimental evidence, we double‐checked the results of YRDC expression. As the same as the previous study has shown, YRDC was highly expressed in lung cancer and hepatocellular carcinoma, and the knockdown and overexpression model validated the expression and protein level of YRDC in cell lines [9, 10]. Overall, YRDC was overexpressed in most of the different types of tumors, which might reveal *YRDC* as a common oncogene.

On the basis of multi‐omics data, we found that the high expression of YRDC in tumor tissues may be the result of *YRDC* CNV and aberrant methylation, which explains part of the phenomenon of highly expressed YRDC in cancer. We further screened the potential transcription factors such as ERG and GABPA, which might regulate YRDC with the Toolkit application. Previous studies have shown that MicroRNA‐206 regulated bladder cancer development via targeting YRDC [43]. Circular RNA circRBMS3 targeting the YRDC axis regulated the proliferation of osteosarcoma [42].

It was firstly reported that the high level of YRDC resulted in a worse prognosis for patients with 10 tumors (BLCA, BRCA, CHOL, HNSC, KICH, LIHC, LUAD, MESO, SKCM and THCA). The expression profiles in breast invasive carcinoma, cholangiocarcinoma, head and neck squamous cell carcinoma, liver hepatocellular carcinoma, and lung adenocarcinoma were consistent with prognostic values. In COAD and KIRC patients, overexpressed YRDC independently predicted a better prognosis.

According to our results, YRDC is likely to promote tumors. Huang et al. reported that in bladder cancer, the blocking of YRDC caused inhibition of bladder cancer cell proliferation, colony formation, migration, and invasion. In addition, restoration of YRDC partially reversed the effects on bladder cancer cells [43]. A recent study reported that activation of the MEK/ERK pathway by YRDC promoted the proliferation of hepatocellular carcinoma [10]. Through co‐expression analysis and GSEA analysis, in our study, YRDC might accelerate cell proliferation in various tumors, which was consistent with previous studies. Our results also implicated that tumor cells might acquire drug resistance due to the high level of *YRDC*. High levels of YRDC caused tumor progression by accelerating cell proliferation and drug resistance. Modulation of YRDC translation promoted resistance to EGFR‐TKI [44]. Additionally, through co‐expression analysis and GSEA, we observed associations between YRDC and mTOR and PI3K‐Akt pathways across multiple tumors. Given the known relevance of these pathways to tumor progression [45, 46], we speculate that YRDC likely influences tumor proliferation and drug resistance through those pathways.

Using the co‐expression analyses, YRDC was related to immune cell infiltration. We found that high YRDC expression closely correlated with low levels of infiltration of CD8+ T cells, memory B cells, plasma cells, and activated NK cells. The CD8‐positive T cells possess cytotoxic molecules, including granzymes and perforin, which can kill tumor cells [47]. Plasma cells, developing from B cells, produce antibodies which are essential components in antitumor immunity. Moreover, GSEA analysis revealed that YRDC was related to deregulating the interferon‐alpha response pathway. Previous studies have shown that CD8+ T cells could be enhanced upon IFN‐α costimulation [48]. Besides, we observed that the expression level of PD‐L1 and PD‐L2 was linked with YRDC. The combination of PD‐1 with PD‐L1/PD‐L2 restrains antitumor immunity by modulating T‐cell activity and activating T cell apoptosis [49, 50, 51, 52]. Moreover, the public cohort confirmed that the high level of YRDC results in a worse prognosis for patients with immune checkpoint inhibitor treatment. Thus, the expression of YRDC has a favorable prognostic value for the response to immune checkpoint inhibitors.

The identification of YRDC as a potential prognostic marker and a risk stratification marker opens up new avenues for improving patient outcomes. For instance, elevated levels of YRDC might indicate aggressive tumor behavior in breast invasive carcinoma, cholangiocarcinoma, head and neck squamous cell carcinoma, liver hepatocellular carcinoma, and lung adenocarcinoma. It might give hints to clinicians that those five tumor patients with overexpressed YRDC stratified as high‐risk groups should receive more aggressive treatment regimens and more frequent follow‐ups. What is more, understanding the role of YRDC in immune cell infiltration and its interaction with other molecular pathways can offer a novel personalized therapeutic strategy, such as developing specific inhibitors targeting YRDC. And we also found high YRDC levels were associated with poor treatment efficacy of immune checkpoint inhibitors. Using YRDC genetic profiling of tumors could help identify patients who would benefit most from immune checkpoint inhibitors, thereby optimizing treatment outcomes.

We presented a bioinformatics analysis of the possible pro‐tumor molecular mechanisms and biomarker role of YRDC. Several limitations should be noted in our study. Firstly, further experimental validation is needed to confirm and explore functional roles of YRDC. Secondly, the sample sizes for some cancer types were relatively small, which may affect the generalizability of our results. Future studies should investigate YRDC's mechanisms using in vitro and in vivo models, as well as validate its clinical utility in larger patient cohorts.

## 5 Conclusions

In summary, YRDC was suggested as a potential innovative biomarker for tumor patients prognosis, immunotherapy response, and targeted drug's efficacy. Additionally, it provided a comprehensive analysis of YRDC's molecular mechanisms at a pan‐cancer level, which can help to further investigate its potential mechanisms in tumorigenesis and malignant progression.

## Author Contributions

P.H. designed the study, and all authors performed the data analyses, contributed to the conception of the study and drafted the manuscript, and contributed significantly to writing the manuscript.

## Ethics Statement

The authors have nothing to report.

## Conflicts of Interest

The authors declare no conflicts of interest.

## Supporting information


**Figure S1:** Expression levels of YRDC in different pathological stages.

**Figure S2:** Outcomes (OS) of patients with different YRDC expressions.

**Table S1:** List of the tumors involved in each analysis.

## Data Availability

The data that support the findings of this study are available in TCGA at https://portal.gdc.cancer.gov/. These data were derived from the following resources available in the public domain: dpGaP, https://www.ncbi.nlm.nih.gov/gap/.
