# Supplementary figures and images for: YRDC is a Prognostic‐Related Biomarker Correlated With Immune Infiltration and Drug Sensitivity in Pan‐Cancer
Source: Cancer Rep (Hoboken). 2025 Sep 2;8(9):e70325. doi: 10.1002/cnr2.70325 (PMC12405056; doi:10.1002/cnr2.70325)

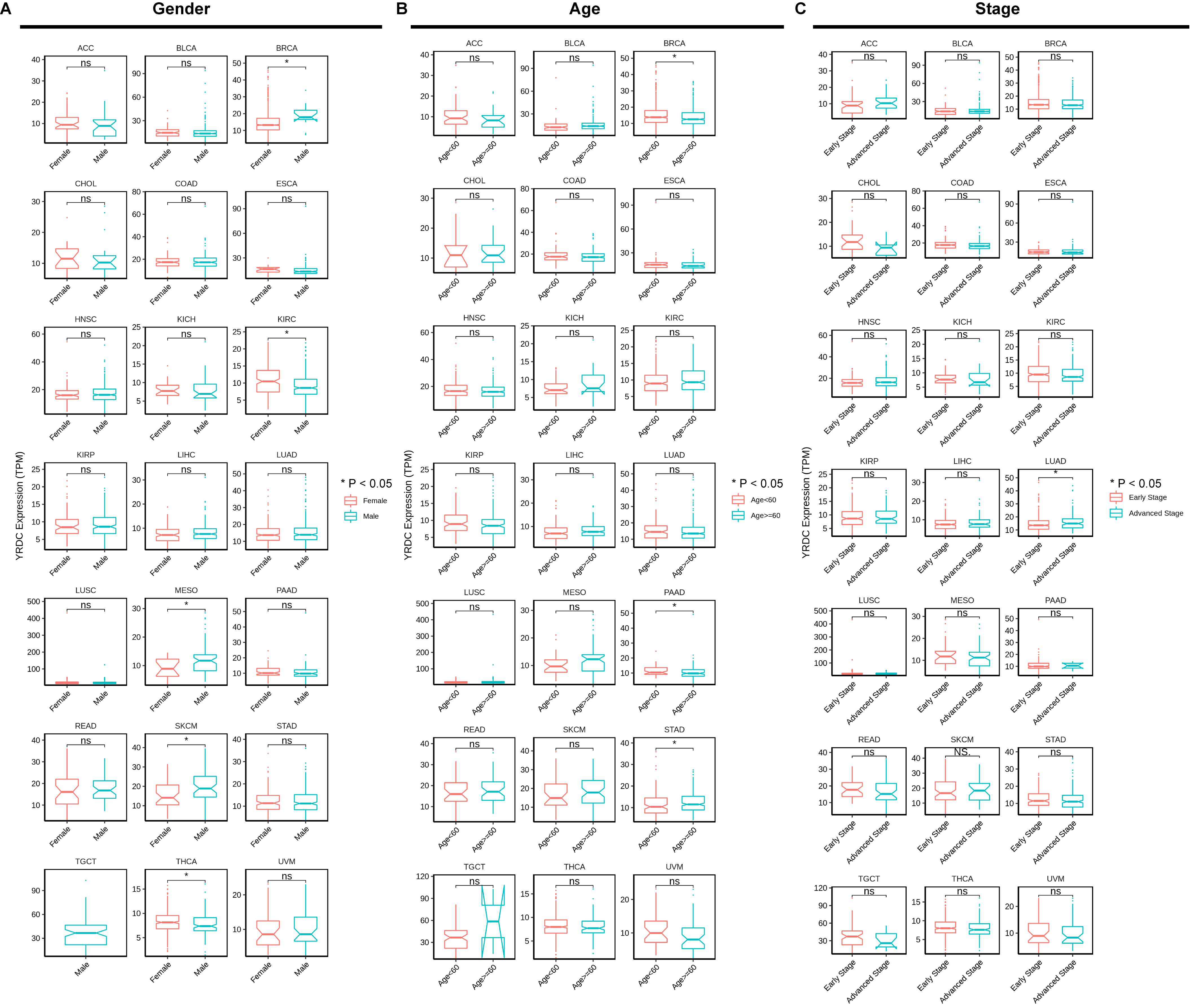

Supplement: Supplementary file 1 — Figure S1: Expression levels of YRDC in different pathological stages. [file CNR2-8-e70325-s001.tiff]

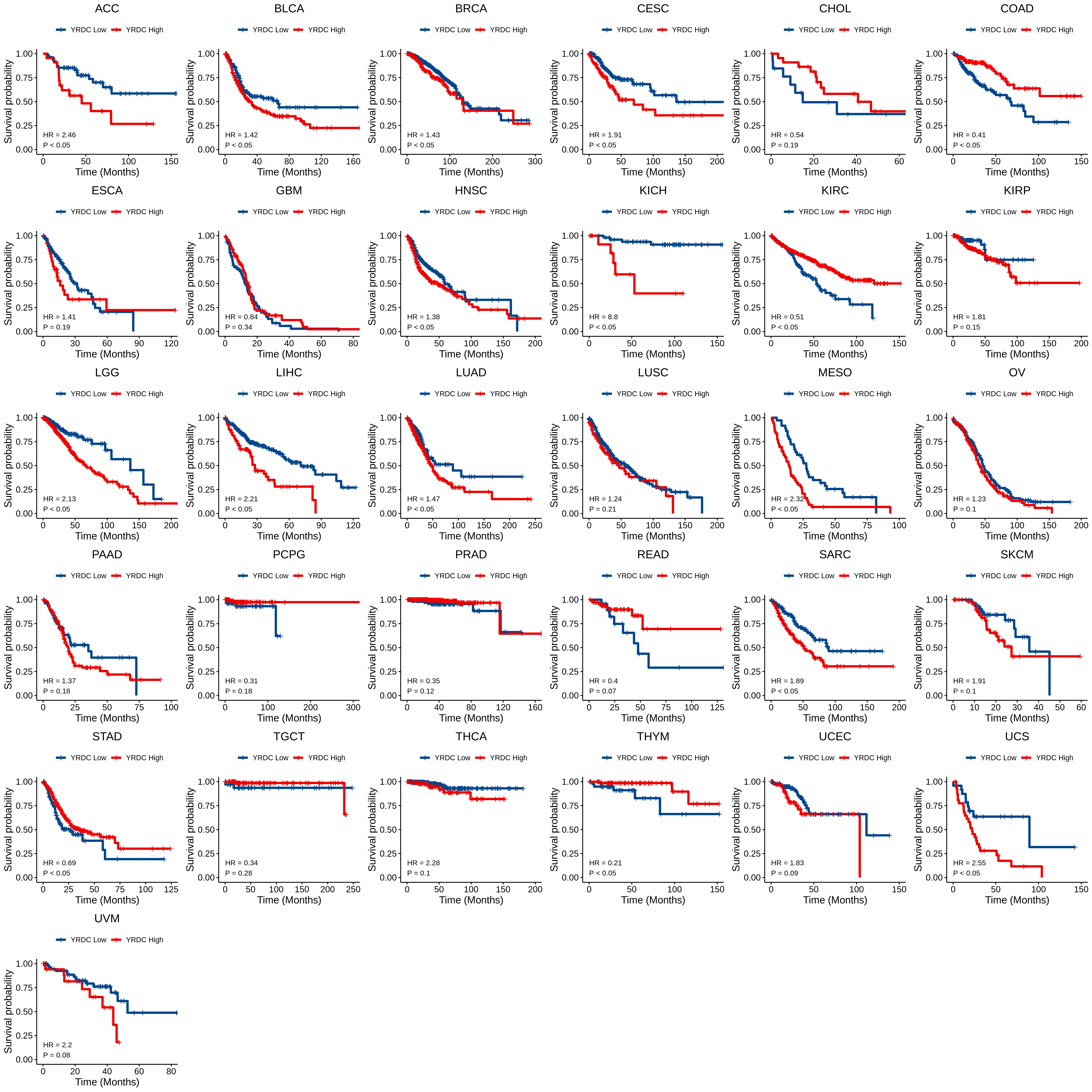

Supplement: Supplementary file 2 — Figure S2: Outcomes (OS) of patients with different YRDC expressions. [file CNR2-8-e70325-s002.tiff]
